# Use of Telecommunication and Diabetes-Related Technologies in Older Adults With Type 1 Diabetes During a Time of Sudden Isolation: Mixed Methods Study

**DOI:** 10.2196/38869

**Published:** 2022-11-18

**Authors:** Elena Toschi, Christine Slyne, Katie Weinger, Sarah Sy, Kayla Sifre, Amy Michals, DaiQuann Davis, Rachel Dewar, Astrid Atakov-Castillo, Saira Haque, Stirling Cummings, Stephen Brown, Medha Munshi

**Affiliations:** 1 Joslin Diabetes Center Boston, MA United States; 2 Beth Israel Deaconess Medical Center Boston, MA United States; 3 Harvard Medical School Boston, MA United States; 4 Research Triangle Institute International Research Triangle Park, NC United States

**Keywords:** type 1 diabetes, older adults, COVID-19, diabetes technology, continuous glucose monitoring, telehealth, diabetes, glucose monitoring, older population, health technology

## Abstract

**Background:**

The COVID-19 lockdown imposed a sudden change in lifestyle with self-isolation and a rapid shift to the use of technology to maintain clinical care and social connections.

**Objective:**

In this mixed methods study, we explored the impact of isolation during the lockdown on the use of technology in older adults with type 1 diabetes (T1D).

**Methods:**

Older adults (aged ≥65 years) with T1D using continuous glucose monitoring (CGM) participated in semistructured interviews during the COVID-19 lockdown. A multidisciplinary team coded the interviews. In addition, CGM metrics from a subgroup of participants were collected before and during the lockdown.

**Results:**

We evaluated 34 participants (mean age 71, SD 5 years). Three themes related to technology use emerged from the thematic analysis regarding the impact of isolation on (1) insulin pump and CGM use to manage diabetes, including timely access to supplies, and changing Medicare eligibility regulations; (2) technology use for social interaction; and (3) telehealth use to maintain medical care. The CGM data from a subgroup (19/34, 56%; mean age 74, SD 5 years) showed an increase in time in range (mean 57%, SD 17% vs mean 63%, SD 15%; *P*=.001), a decrease in hyperglycemia (>180 mg/dL; mean 41%, SD 19% vs mean 35%, SD 17%; *P*<.001), and no change in hypoglycemia (<70 mg/dL; median 0.7%, IQR 0%-2% vs median 1.1%, IQR 0%-4%; *P*=.40) during the lockdown compared to before the lockdown.

**Conclusions:**

These findings show that our cohort of older adults successfully used technology during isolation. Participants provided the positive and negative perceptions of technology use. Clinicians can benefit from our findings by identifying barriers to technology use during times of isolation and developing strategies to overcome these barriers.

## Introduction

The use of technology for diabetes management, as well as for communication and social interaction, has become more prevalent over the last decade. However, older adults may be less proficient and equipped to use technology compared to younger generations [1]. In addition, older adults may experience physical and cognitive decline during social isolation and periods of being homebound [2,3], which may further impact their ability to use technology.

Older adults with type 1 diabetes (T1D) are a unique population with challenges related to the management of their diabetes [4-6]. Many of them rely on the use of diabetes-related technologies, such as insulin pumps and continuous glucose monitoring (CGM), to manage diabetes on a daily basis.

The use of insulin pumps and CGM devices has proven to be beneficial in older adults in improving glycemic control and reducing hypoglycemia [7,8]. However, the use of diabetes-related technologies in older adults with T1D is lower than in younger adults with T1D [9,10].

The COVID-19 lockdown triggered a sudden and dramatic change in routine, with self-isolation and a rapid shift to the use of technology for maintaining clinical care and social connections [11]. The lockdown offered a unique opportunity to assess how older adults fared with technology for diabetes management, communication, and social interaction during a time of sudden isolation.

During the lockdown, an ongoing study of older adults with T1D using CGM was paused, providing an important opportunity to examine how isolation affects older adults with T1D using technology. We performed interviews with participants of the study to understand the positive and negative perceptions of technology use in this population during times of isolation. In addition, we examined glucose parameters via CGM in a subpopulation of this cohort with available data to understand the impact of isolation on glycemic control both before and during this period.

## Methods

### Study Design

We performed semistructured interviews with 34 participants from the ongoing study titled “Technological Advances in Glucose Management in Older Adults,” which assessed the use of CGM in older adults with T1D (Clinicaltrials.gov NCT03078491). Interviews were performed between May and August 2020. Each interview lasted 30-60 minutes and was conducted via phone, digitally audio-recorded, and transcribed. Interviews included 4 broad questions with probes, including (1) How are you doing during the COVID-19 pandemic? (2) How are you managing your diabetes during this COVID-19 pandemic? (3) How are you doing using your diabetes technology? and (4) Have you noticed any changes in your emotions during this time? and 23 survey questions. Eligibility criteria for these interviews included enrollment in the “Technological Advances in Glucose Management in Older Adults” study and willingness and capability to participate. All participants were wearing real-time CGM devices and provided verbal informed consent over the phone.

The interviews were later transcribed, coded using NVivo software (version 12; QRS International), and analyzed using qualitative content analysis by categorizing keywords and phrases to identify themes. We achieved investigator triangulation [12], a process in which more than 1 investigator analyzes the data, through the use of a multidisciplinary team with members experienced in the care of older adults with diabetes. Members included a geriatrician, an endocrinologist, a health informaticist, a psychologist and nurse educator, and research assistants. Interviews were individually coded and then members met via teleconference over a 6-month period to establish group consensus regarding the identification and definition of themes and the selection of examples from transcripts, as well as the status of data saturation. In the results section of this manuscript, the age, gender, and diabetes duration of the participants are provided for each quotation. All names of clinical providers, medical supply companies, and medications and brand names of device companies were omitted and replaced by either initials or generic names within quotations.

CGM data from both before and during isolation were available for 19 (56%) of the 34 individuals interviewed, which included a 2-week period between January and February 2020 (preisolation) and a 2-week period between April and June 2020 (during isolation). A minimum of 192 hours per week of CGM data were required for inclusion in this CGM analysis. CGM metrics, including total percent time in range (defined as total percent time spent between 70 and 180 mg/dL), total percent time spent in hypoglycemia (defined as total percent time spent below 70 mg/dL), total percent time in hyperglycemia (defined as total percent time spent above 180 mg/dL), and coefficient of variation were analyzed. The coefficient of variation (%) was calculated as *(SD of glucose / mean glucose level) × 100* [13].

Descriptive statistics for demographic and clinical data are reported as number (n) and percentage (%) of the cohort for categorical variables. For continuous variables, data are reported as mean (SD) for data with normal distributions and as median (IQR) for data with nonnormal distributions. SAS software (version 9.4; SAS Institute) was used for 2-tailed Student *t* tests for the analysis of CGM metrics. A *P* value of ≤.05 was considered statistically significant.

### Ethics Approval

The Joslin Diabetes Center Institutional Review Board approved the study protocol (CHS #2016-29).

## Results

### Participant Demographics

In all, 34 participants, with a mean age of 71 (SD 5) years and a mean duration of diabetes of 30 (SD 17; range 10-65) years, were interviewed (Table 1).

**Table 1:** Demographics and clinical characteristics of the study population.

Characteristic					Value (N=34)
**Demographics**					
	Age (years), mean (SD; range)			70.9 (4.8; 66-86)	
	Gender, female, n (%)			18 (53)	
	Ethnicity, White, n (%)			33 (97)	
	**Marital status, n (%)**				
		Single	10 (29)		
		Married	23 (68)		
	**Living status, n (%)**				
		Private home	18 (53)		
		Apartment or condo	15 (44)		
	Currently working, n (%)			8 (24)	
	Some college or higher level education, n (%)			32 (94)	
**Diabetes characteristics**					
	Using a real-time continuous glucose monitor, n (%)			34 (100)	
	Using an insulin pump, n (%)			20 (59)	
	Diabetes duration (years), mean (SD; range)			38.0 (17.4; 10-65)	
	Hemoglobin A_1c_ (%), mean (SD)			7.4 (0.93)	
	**Mode of engagement with social networks, n (%)**				
		Video calls	18 (53)		
		Phone calls	14 (41)		
	**Connecting with loved ones, n (%)**				
		More than usual	13 (38)		
		Less than usual	18 (53)		
		The same as usual	14 (41)		
	Had contact with their primary care provider, n (%)			22 (65)	
	Confident medical needs are being met, n (%)			28 (82)	
	Had a telehealth visit at the time of interview, n (%)			24 (71)	

### CGM Data

We analyzed the CGM data collected from a 2-week period between January and February 2020 (preisolation) and a 2-week period between April and June 2020 (during isolation) from a subpopulation of the study cohort. We analyzed data from 19 (N=34, 56%) participants (mean age 74, SD 5 years; 11/19, 58% female; 12/19, 63% insulin pump users; and mean diabetes duration 38, SD 17, range 14-69 years). The CGM metrics showed that during the lockdown, compared to before the lockdown, percent time spent in range increased (mean 57%, SD 17% vs mean 63%, SD 15%; *P*=.001) and percent time spent in hyperglycemia (glucose >180 mg/dL) decreased (mean 41%, SD 19% vs mean 35%, SD 17%; *P*<.001). These changes resulted in a reduction of glucose management indicator (mean 7.5%, SD 0.7% vs mean 7.2%, SD 0.6%; *P*=.003) during the lockdown. Our cohort had very little hypoglycemia, which did not change from before to during the lockdown (median 0.7%, IQR 0%-2% vs median 1.1%, IQR 0%-4%; *P*=.40; Table 2).

**Table 2:** CGM^a^ metrics from a 2-week period between January and February 2020 (preisolation) and a 2-week period between April and June 2020 (during isolation; n=19). Data are presented as mean (SD) for data with normal distributions and as median (IQR) for data with nonnormal distributions.

CGM metrics	Preisolation	During isolation	*P* value
Time spent in hypoglycemia <55 mg/dL (%), median (IQR)	0.1 (0-1)	0.1 (0-2)	.40
Time spent in hypoglycemia <70 mg/dL (%), median (IQR)	0.7 (0-2)	1.1 (0-4)	.40
Time spent in the range of 70-180 mg/dL (%), mean (SD)	57 (17)	63 (15)	.001
Time spent >180 mg/dL (%), mean (SD)	41 (19)	35 (17)	<.001
Time spent >250 mg/dL (%), mean (SD)	12 (11)	9 (8)	.08
Glucose management indicator (%), mean (SD)	7.5 (0.7)	7.2 (0.6)	.003
Coefficient of variation (%), mean (SD)	33 (5)	33 (5)	.86

^a^CGM: continuous glucose monitoring.

### Themes

Next, we identified 3 themes regarding the positive and negative perceptions related to the use of technologies for diabetes management, social interactions, and medical care (Table 3).

**Table 3:** Themes and subthemes regarding the impact of isolation during pandemic lockdown on technology use.

Theme, content area		Patient perspective
**Use of diabetes-related technologies (pump and CGM^a^)**		
	Use of CGM to manage diabetes	Able to monitor glucose Peace of mind, especially overnight Alarms to help with diabetes management Able to share data with clinicians Challenging to keep track of supplies Fearful of not having sufficient CGM supplies to be able to use CGM device at all times
	Use of insulin pump to manage diabetes	Comfortable adjusting insulin pump settings to address changes in insulin requirements during times of change in daily activity Concerns about changing to new a device during a challenging time
	Diabetes-related medical supplies	Concerns about receiving supplies in a timely fashion Appreciated change in Medicare policies to maintain continuity of care Difficulties in communication with third party suppliers Frustration with the limited availability of supplies Delays in shipment
**Use of technology for social interaction**		
	Social gathering	Help with connecting to family and friends and participation in courses, clubs, and support groups Lack of in-person interaction
**Use of technology for medical interaction**		
	Telehealth	Able to connect with medical team Time saving Delays in the coordination of care Lack of physical exam and laboratory data
	Usual care	Provided continuity of care Experienced delays in care for interventional medicine, such as dental care and eye exam

^a^CGM: continuous glucose monitoring.

#### Theme 1: Impact of Isolation on the Use of Insulin Pump and CGM to Manage Diabetes, Including Timely Access to Supplies, and Changing Medicare Eligibility Regulations

All participants, without fail, had positive perceptions of using CGM during isolation. Several participants reported that using CGM to manage diabetes during a time of lifestyle change provided reassurance: “I rely on [my CGM] and I’m having it help me get through the night particularly” (72-year-old woman, T1D for 14 years). In addition, the built-in alarms were very important, said one participant: “I forget to take [my insulin]...my CGM is buzzing” (87-year-old man, T1D for 58 years).

A majority (28/34, 82%) of participants reported feeling confident in managing their glucose patterns, even during this time of disruption in daily life, because of CGM.

Once in a while my blood sugar would go high or low, but not high or low enough to cause any concerns. Sometimes I might forget to take a bolus. So, it might go high, but with the CGM I'm able to take a look and see what's happening and then I can correct for it.

68-year-old man, T1D for 44 years

A few participants felt that CGM could be helpful to detect a COVID-19 infection early by showing high glucose readings. One participant noted, “I’m going to assume that if I had a virulent virus, that my blood sugars may easily be affected. If that in fact were the case, I’m speculating now, it—the CGM—would provide an additional level of comfort” (68-year-old man, T1D for 44 years).

Among the participants using insulin pumps (20/34, 59% of this cohort), many reported being comfortable in adjusting insulin pump settings to address changes in insulin requirements during times of change in daily activity: “Actually, it’s better because I’ve managed to adjust the pump now...I’ve been able to deal with the pump settings a lot more easily” (72-year-old man, T1D for 33 years).

However, a major change in pump therapy might be problem, as one person who was planning to change to a different type of pump device reported:

I’ve been in touch with my doctor, and I’ve been in touch with the educators, and we’re looking at a new pump. I’m a little concerned about how that’s going to work, because there are some things about it that are concerning to me. And I just don’t know if I am in the frame of mind to face a new challenge.

69-year-old woman, T1D for 49 years

In addition to the benefits of CGM and insulin pump use, participants reported that the ability to share data from these devices with their providers was helpful to guide conversation during medical visits: “Dr. T had me download the [CGM data]...And then, she got those results. And so, we talked about those, and so forth. And I had it in front of me, and she had it. And so, that went well” (75-year-old woman, T1D for 28 years). Another participant said, “These visits, you don’t really have to be there. It’s really a question of talking and reviewing things and answering questions. Telemedicine lends itself very well to that, I think. I was very satisfied we talked” (72-year-old man, T1D for 33 years).

However, many participants worried about the potential loss of CGM supplies, because they rely heavily on CGM data to manage their diabetes. For example, one woman said, “I would say, if you really wanted to get me upset and afraid, take my CGM away. So, I am very dependent on it” (71-year-old woman, T1D for 14 years). There were also concerns related to supply chain, third party suppliers, and insurance companies regarding timely paperwork processing and supplies shipment during lockdown: “I used to call up and they would say you only get a certain number. They really send me one in a box or something. And then all of a sudden, I’m getting a box of three” (72-year-old woman, T1D for 53 years). Another participant said:

Insurance has made it extremely difficult to have it work smoothly for getting your supplies. So I’m a little apprehensive that, now that I’ll be getting supplies, it looks like, from two different companies and not directly through [pump company] and [CGM company]. And when you have to depend on two supply companies shipping you supplies, and especially after what happened with this virus, you know, shipping isn’t what it was.

76-year-old woman, T1D for 53 years

A few participants also worried that as they get older, they will have more difficulty keeping up with the processes and regulations of the complex supply system. For example, one person said:

And now with [CGM], I’m down to my last sensor kit. So I’m finishing the one that I have up, I think this weekend. And so, then I have to order the next one, then make sure they get that shipped out. Like I say, right now I’ve got my faculty, but 20 years from now, who knows?

77-year-old man, T1D for 18 years

Additionally, many participants expressed concerns during the interview regarding adhering to Medicare regulations, as these regulations constantly changed during the pandemic, and participants were not always informed: “With Medicare, you have to be seen every three months and you have to have an A_1c_ done. And if these doctor’s offices have been either closed or they don’t call you back and so just trying to coordinate my care has been the biggest issue” (76-year-old woman, T1D 53 years). Some expressed frustration with Medicare rules, overall: “The visit to a doctor every three months, in my opinion, is a waste of money...I talked to the Medicare man about getting the supplies, and he said, ‘Well, the doctor visit is important. We want to make sure that your diabetes hasn’t gone away’” (68-year-old woman, T1D for 51 years).

#### Theme 2: Impact of Isolation on Technology Use for Social Interaction

The second theme identified was the use of technology as a tool for maintaining social life and connecting with friends and family. More than half (18/34, 53%) of the participants used video call as a way to connect with others and 38% (13/34) reported to be in touch with others more frequently than before lockdown (Table 1). One woman said, “I’m taking courses and things like that. I’m doing some Zoom get-togethers” (72-years old, T1D for 20 years). Another person noted, “we’ve used Zoom to get together with our kids and family, so we do a family Zoom meeting. And the kids are always calling and family FaceTime with our grandkids” (72-year-old man, T1D for 65 years). Many participants reported they were able to maintain communication and attend religious functions: “I’m playing mahjong with friends online. We go to services from a synagogue online...it’s good to interact” (69-year-old woman, T1D for 49 years). One person reported that she was able to interact remotely using a web-based platform to help out while her daughter worked: “With my granddaughter, oh, we have so much fun. We do FaceTime. I actually call it babysitting” (72-year-old woman, T1D for 20 years).

There was also an increased opportunity to participate in a support group for people using insulin pumps, with an even broader reach than in-person meetings: “I’m part of an insulin pump support group...We’ve actually attracted additional people that not only don’t show up normally for the monthly meetings, but we’ve also gone outside this geographic area” (76-year-old man, T1D for 67 years).

However, other participants reported giving up some of their regular activities, such as playing chess or religious meetings, due to the lack of in-person interactions: “There’s a lot of church groups that you have Zoom meetings, and that’s awkward. I’d rather be meeting in-person instead of Zoom” (71-year-old woman, T1D for 30 years). In fact, a frequent complaint in this cohort of older adults was the lack of physical interaction with their grandchildren: “We have children. We have grandchildren. We’re unable to be around them, and that’s heartbreaking in a lot of ways. We like to see them” (69-year-old woman, T1D for 49 years). Another person said:

So it’s a whole different thing to worry about when you’re talking with [grandkids] on the phone. How do I engage and help them without being able to sit in the room with them? I have another grandchild...who just had his first birthday party on Zoom. It sucks.

69-year-old man, T1D for 49 years

#### Theme 3: Impact of Isolation on Telehealth Use to Maintain Medical Care

Telemedicine was rapidly implemented at the beginning of the COVID-19 lockdown to provide care when in-person visits were not allowed. At the time the interviews occurred, 71% (24/34) of the participants already had at least one video visit, 65% (22/34) had contact with their primary care provider, and 82% (28/34) reported feeling confident that their medical needs were met **(**Table 1)**.**

Many of the participants reported that telemedicine was adequate and the delivery of care via telemedicine was able to address their needs. One participant said, “I had [a telemedicine visit] with my allergist and also with my primary care physician, and I came loaded with questions, and I had them all answered. Even if I didn’t like the answers. So the quality of care was good” (72-year-old man, T1D for 65 years). Others voiced that their telemedicine visit with their diabetes team was as good as an in-person visit and may be time- and cost-saving.

If it’s not a visit to get blood work done, I’d definitely have a conversation over video chat...I like it, because number one, I don’t have to drive into Boston with all the traffic...I think this is a great tool and I’m glad, in a positive way, that COVID actually got this up and running.

69-year-old woman, T1D for 29 years

I had a urinary tract infection and I had a phone conference with my urologist, which I felt was adequate. And treatment was, I don’t think would have been any different than if I sat in his office and waited for 40 minutes, and then spent five minutes with him in person.

68-year-old man, T1D for 42 years

However, some participants reported the lack of physical interactions and laboratory data as major drawbacks of telemedicine. One man said, “I think these telemeetings...[My provider] couldn’t take blood pressure, or anything like that...And I’m thinking, ‘Yeah, well, that’s not very useful’” (72-year-old man, T1D for 31 years). Another man said, “One of the things they’re supposed to do is check my feet...They want to see how my balance is when I’m walking or how stable I am...I just don’t have those words and terminology to relay the information to them” (69-year-old man, T1D for 49 years). One person reported, “I really don’t see any value for me personally, in telemedicine. Unless I’m having a real problem, I feel like I can manage the diabetes myself...in my particular situation, I didn’t opt for any telemedicine visits” (76-year-old man, T1D for 67 years).

Some participants voiced concerns regarding remote visits for diabetes management due to their inability to change insulin pump settings without hand-on assistance from their provider.

I think I really prefer seeing my diabetes caregivers in person and making changes to my treatment, my CGM and my pump because I still don’t feel capable of making those changes myself when things are not going smoothly, and that’s a worry.

79-year-old woman, T1D for 58 years

COVID-19 imposed delays in medical care requiring in-person visits, such as surgery, dental care, and eye care, which telemedicine could not address. One woman noted, “I should’ve had a cochlear implant surgery. And that’s been put on hold” (72-year-old woman, T1D for 60 years). Another woman said, “my dental stuff, I’m not in any pain or anything but I knew I needed to do that at some point, so the COVID is standing in my way, in part, for that” (73-year-old woman, T1D for 24 years). “I was due for an eye exam and that was put on hold” (76-year-old woman, T1D for 53 years), stated one woman.

## Discussion

This study shows that older adults with T1D were able to continue to use diabetes-related technologies, such as insulin pump and CGM, during a time of isolation, to maintain their diabetes management. However, the participants described barriers to and enablers of technology use that have not yet been described in the literature. Although pandemics are rare, sudden isolation can occur in the lives of older persons due to the loss of a significant other, acute illness, or decline in cognitive or functional status. Understanding how older persons with T1D interact with technologies may help clinicians to develop age-specific pathways to support this unique population during times of sudden isolation.

All of the participants in our study voiced that the use of CGM was beneficial during the lockdown. This finding is important, considering that the adoption of CGM in older adults has lagged behind the adoption in the younger population. A recent study of a large cohort from the T1D Exchange (2016-2018) showed that only 34% of adults aged >50 years with T1D were using personal CGM [14]. A majority (22/34, 65%) of our cohort in this study had strong positive perceptions about the benefits of readily available data and alarms from CGM. Participants reported that being able to share CGM data with clinicians during telemedicine visits was helpful to assess glucose patterns (time in range and time spent in hyperglycemia and hypoglycemia). Such information can be very valuable during a remote visit, to provide actual information on glycemic control, when laboratory data, such as Hemoglobin A_1c_, are unavailable [15]. Furthermore, the CGM metrics collected in a small subgroup of participants did not show a worsening of glycemic control during a lockdown, which is consistent with other reports in adults with T1D [16]. Thus, all of these findings, taken together, support the benefits of CGM use in older adults with T1D, even during a time of isolation.

We also found that the reliability of timely access to supplies for CGM and insulin pump was concerning for many of the participants. Most participants reported some concerns with shipment arrival and the quantity of supplies shipped. This issue was a nationwide problem at the beginning of the pandemic when shipping companies got overwhelmed by the increased volume of shipment [17]. Fortunately, only a few people experienced actual delivery delays; however, their anxiety remained a concern. Similarly, until the COVID-19 lockdown, Medicare required patients with T1D to be seen in-person every 3 and 6 months for pump and CGM, respectively. Medicare addressed and modified the rules very early in the pandemic; however, not all participants were aware of these changes. These findings highlight the issues older adults face with accessing current regulatory and administration information with the use of diabetes technology. In addition to assistance with the use of technology, this population would benefit from structured assistance with regulatory and administrative tasks.

The use of technology to communicate with others is a recent advancement and not all older adults are proficient in its use [1]. Most of our interviewees felt that communication technology was helpful to keep in contact with family and friends. Many of the participants in our cohort reported enrolling in new social events held remotely during the COVID-19 lockdown, such as book clubs, support groups, or happy hours. These findings are consistent with another population-based representative survey conducted in older adults during the COVID-19 lockdown, showing their ability to use technology to mitigate social isolation [18]. However, several participants reported missing physical contact with their young grandchildren and were not engaged in remote socialization. Our findings further highlight the results from a recent study showing persistent loneliness in older adults who face barriers to technology-based social interactions [19]. Increasing isolation in older adults during COVID-19 has been associated with a worsening in mental and physical health in some studies [20]. Thus, assisting older adults with T1D to overcome barriers to communication technology use during isolation and promoting in-person interaction as much as possible is an important intervention to maintain their mental health.

The majority of our participants voiced that the use of web-based technologies for telemedicine lessened the negative impact of isolation on their health care. Several recent studies have shown that telehealth visits have been as beneficial as in-person visits to manage diabetes in people with both T1D and type 2 diabetes [16,21,22]. The CGM data from our cohort support these findings. However, in-person visits remain important for subspecialties such as dentistry, podiatry, and ophthalmology, as well as laboratory data for routine clinical care. Overall, our study supports the benefits of the use of telemedicine for older persons with T1D using CGM for the management of diabetes.

The limitations of the study included the homogeneity of our participants. Almost all participants are non-Hispanic White, with high levels of education, and universally use CGM; thus, our results may not be generalizable to all older adults with T1D.

In conclusion, our cohort of older adults with T1D using CGM were able to use technologies to maintain their diabetes management, social interactions, and medical care during isolation. The participants provided both the positive and negative perceptions of technology use, which can help clinicians identify barriers to technology use and strategies to overcome these barriers in their patient population during isolation from any cause.

## Abbreviations

CGM: continuous glucose monitoring
T1D: type 1 diabetes
